# Energy expenditure in chronic stroke patients playing Wii Sports: a pilot study

**DOI:** 10.1186/1743-0003-8-38

**Published:** 2011-07-14

**Authors:** Henri L Hurkmans, Gerard M Ribbers, Marjolein F Streur-Kranenburg, Henk J Stam, Rita J van den Berg-Emons

**Affiliations:** 1 Department of Rehabilitation Medicine and Physical Therapy, Erasmus MC - University Medical Center, P.O. Box 2040, 3000 CA Rotterdam, The Netherlands; 2 Rijndam Rehabilitation Centre, P.O. Box 2040, 3000 CA Rotterdam, the Netherlands

## Abstract

**Background:**

Stroke is one of the leading causes of long-term disability in modern western countries. Stroke survivors often have functional limitations which might lead to a vicious circle of reduced physical activity, deconditioning and further physical deterioration. Current evidence suggests that routine moderate- or vigorous-intensity physical activity is essential for maintenance and improvement of health among stroke survivors. Nevertheless, long-term participation in physical activities is low among people with disabilities. Active video games, such as Nintendo Wii Sports, might maintain interest and improve long-term participation in physical activities; however, the intensity of physical activity among chronic stroke patients while playing Wii Sports is unknown. We investigated the energy expenditure of chronic stroke patients while playing Wii Sports tennis and boxing.

**Methods:**

Ten chronic (≥ 6 months) stroke patients comprising a convenience sample, who were able to walk independently on level ground, were recruited from a rehabilitation centre. They were instructed to play Wii Sports tennis and boxing in random order for 15 minutes each, with a 10-minute break between games. A portable gas analyzer was used to measure oxygen uptake (VO_2_) during sitting and during Wii Sports game play. Energy expenditure was expressed in metabolic equivalents (METs), calculated as VO_2 _during Wii Sports divided by VO_2 _during sitting. We classified physical activity as moderate (3-6 METs) or vigorous (> 6 METs) according to the American College of Sports Medicine and the American Heart Association Guidelines.

**Results:**

Among the 10 chronic stroke patients, 3 were unable to play tennis because they had problems with timing of hitting the ball, and 2 were excluded from the boxing group because of a technical problem with the portable gas analyzer. The mean (± SD) energy expenditure during Wii Sports game play was 3.7 (± 0.6) METs for tennis and 4.1 (± 0.7) METs for boxing. All 8 participants who played boxing and 6 of the 7 who played tennis attained energy expenditures > 3 METs.

**Conclusions:**

With the exception of one patient in the tennis group, chronic stroke patients played Wii Sports tennis and boxing at moderate-intensity, sufficient for maintaining and improving health in this population.

## Background

Stroke is one of the leading causes of long-term disability in modern western countries [1]. As a consequence of European population aging, the number of strokes is predicted to increase from approximately 1.1 million per year in 2000 to 1.5 million per year in 2025 [2]. Worldwide stroke prevalence ranges from 5-10 per 1000 among all age groups and from 46-73 per 1000 among persons aged ≥65 years [3]. There is a growing need for cost-effective treatment for stroke patients, including rehabilitation and tertiary prevention.

Stroke survivors often become deconditioned with an aerobic capacity about half that of age-matched controls [4-6]. Low aerobic capacity compromises functional mobility after stroke [7,8]. This might lead to a vicious circle of physical inactivity and further physical deterioration [4,9]. Mobility status from 1-3 years after stroke significantly deteriorates in 21% of patients, resulting in reduction of activities of daily living, loss of independence, and social isolation [10]. Physical inactivity might also be a risk factor for recurrent stroke and cardiac events by promoting insulin resistance [5,11-13]. Current guidelines, therefore, recommend that routine moderate- or vigorous-intensity physical activity is needed for stroke survivors to improve and maintain their health [4,14]. However, long-term participation in physical activities is low among people with disabilities as a result of person related factors (e.g. reduced mobility, social isolation) and environmental factors (e.g. limited access to stores and buildings, transport, and availability of equipment) [4,15-17].

Active video game (exergame) systems, such as Nintendo Wii Sports, are innovative and potential technologies that might improve daily physical activity levels for persons with chronic physical disabilities. Previous studies reported a mean energy expenditure of 3-4 metabolic equivalents (METs) among able-bodied adults during Wii tennis and boxing [18,19]. This suggests that exergames have the potential to promote and maintain health, according to the American College of Sports Medicine and American Heart Association (ACSM/AHA) Guidelines on physical activity and public health [20]. Practical advantages of exergaming include the ability to train at home with or without online supervision, thus reducing healthcare costs [21]. Furthermore, exergames can provide real-time feedback on performance and progress [22]. They are also enjoyable, and can be performed with able-bodied relatives or friends or in virtual training groups to enhance compliance [22].

Wii Sports is designed for entertainment rather than therapy, which might limit its usability for stroke rehabilitation. However, in a recent pilot study the Wii gaming technology was found to be a safe, feasible and potentially effective alternative to promote motor recovery after stroke [23]. It is unknown, nonetheless, whether Wii Sports is of sufficient intensity (moderate or vigorous) to promote and maintain health in this population. Stroke-specific factors, including elevated muscle tone and postural instability, might have a large demand on oxygen uptake [4,6]. Conversely, these stroke-specific factors might lead to less intense gameplay and consequently lower energy expenditure.

We performed a proof-of-principle pilot study to determine the energy expenditure of chronic stroke patients while playing Wii-Sports. Our hypothesis was that the energy expenditure would indicate moderate- or vigorous-intensity and meet the ACSM/AHA guidelines to improve and maintain health.

## Methods

### Participants

A convenience sample of 10 persons with chronic stroke was recruited from Rijndam Rehabilitation Centre in the Netherlands. Patients were included if they experienced an ischemic infarct ≥ 6 months prior, and were classified as Functional Ambulation Category (FAC) independence level 3, 4 or 5 [24]. Patients with a history of psychiatric disorders or conditions that might influence physical activity and fitness (e.g. lung disease, rheumatoid arthritis) or impair the safety of physical strain (e.g. cardiac disease) were excluded. Additionally, patients were excluded if they could not understand or were unable to perform research tasks as a result of severe cognitive or linguistic disorders or speech barriers, or if they experienced pain in the affected arm and hand. None of the patients were familiar with the Wii before the study. Eligible persons who provided informed consent were included in the study. Patient characteristics were collected from the patient file, including demographics (age, gender), stroke severity using the Bamford scale [25], upper extremity strength and spasticity from the affected side using de Medical Research Council (MRC) scale [26] and the Modified Ashworth Scale [27], balance using the Berg Balance Scale (BBS) [28], and disability based on the Modified Rankin Scale [29]. The protocol was approved by the Medical Ethical Committee of Erasmus MC.

### Instruments

**Figure 1:**
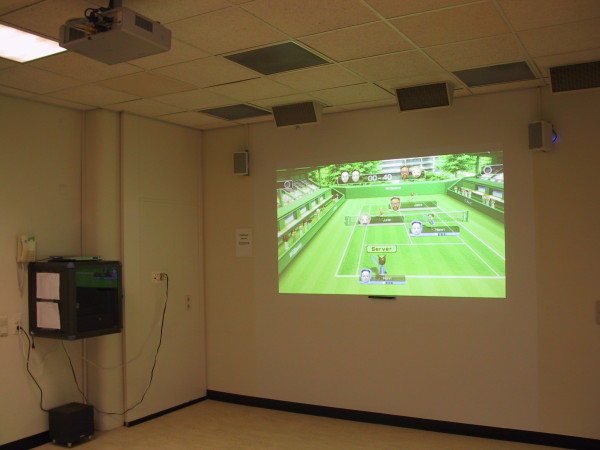
**The Exergame Lab at our department**.

The Nintendo Wii, a home video game console, and the Wii Sports games tennis and boxing were used in the study [30]. The games are played with the Wii remote, which is the primary controller for the console [31]. The Wii remote is a wireless (Bluetooth) device that has a 3-axis accelerometer sensor inside to measure motion in all directions and all speeds. Because of its motion sensing capability, the user is in contact with and can manipulate items on the screen via gesture recognition. For certain Wii games, like Wii boxing, another controller is needed: the Nunchuk. Like the Wii Remote, the Nunchuk also provides a 3-axis accelerometer for motion-sensing and tilting, but without a speaker, a rumble function, or a pointer function. Participants played the Wii games in our department's Exergame Lab, which has a relatively large playing area (5 × 6 meter) with a 1.5 × 2.5 meter beamer projection on the wall along with stereo speakers to provide the visual and audio stimuli (Figure 1).

### Anthropometric and physiologic measurements

Body mass was measured within 0.1 kg accuracy using a calibrated electronic scale (KORONA, Leeds, UK); body height was measured within 0.1 cm accuracy using a wall mounted metal anthropometer (SECA, Hamburg, Germany). Body mass and height were measured with shoes off. Skinfold thickness was measured with a Harpenden Caliper (Burgess Hill, UK) twice on the right side of the body at each of four sites (biceps brachii, triceps brachii, subscapular, and suprailiac). The caliper has a measuring range of 0 to 80 mm, an accuracy of 99%, and a reliability within 0.20 mm. Body fat percentage was calculated according to the equations of Durnin and Womersley [32]. This calculation was then used to determine fat-free mass.

Energy expenditures during game play, sitting, and standing were assessed using a validated portable indirect calorimeter (Cosmed K4b^2^, COSMED, Rome, Italy) [33-38]. Oxygen and carbon dioxide sensors were calibrated with standard gases of known oxygen (16%) and carbon dioxide (5%) concentrations before each Wii tennis and boxing session. A 2-liter volume calibration syringe was used to calibrate the respiratory volume. We measured heart rate (HR) using a Polar T61 heart rate monitor (Polar Electro, Kempele, Finland), which was placed on the participant's chest and connected to the calorimeter. Self-perceived exercise intensity was measured using the modified Borg scale with 0 being "nothing at all" and 10 being "very, very strong" [39,40]. All anthropometric and physiologic measurements were obtained by the same investigator (MF Streur-Kranenburg).

### Experimental trial

Gas exchange measurements were performed during 5 minutes of chair-sitting and during 5 minutes of standing still. Next, participants had up to five minutes to familiarize themselves with the Wii controllers (Wii remote and Nunchuk) and the tennis and boxing games. Then, the participants rested for a minimum of 5 minutes, or until HR had decreased to chair-sitting level.

After resting, the participants played Wii Sports tennis and boxing for 15 minutes each, in random order, with a 10-minute minimum intervening rest period, or until HR had decreased to chair-sitting level. Patients hold the Wii remote in the dominant hand, which could be the affected or non-affected hand. At the conclusion of each tennis match or boxing game, participants restarted the game as quickly as possible and continued to play for a total of 15 minutes. Following each 15 minute game play session, participants rated their perceived exertion using the modified Borg Scale. Participants were allowed to play the game in their own manner and at their own pace. To ensure participant safety and safe handling of measurement equipment, two researchers stood beside the participants during Wii game play.

### Data analysis

Mean (± standard deviation) VO_2 _was calculated for the final 2.5 minutes during sitting and standing, and for the entire 15 minute duration of game play. We calculated energy expenditure, expressed in METs, as the VO_2 _during game play divided by the VO_2 _during sitting. Wilcoxon signed rank tests were used to compare the physiologic variables and perceived exertion measured during Wii tennis with Wii boxing. Wilcoxon signed rank tests were also used to compare physiologic variables measured during game play with those measured during sitting and standing. We used SPSS 16.0 for statistical analyses and set the significance level at P ≤ 0.05.

## Results

Five participants had a maximum score of 5 on the FAC, indicating an ability to ambulate on non-level and level surfaces, stairs, and inclines, one of whom used an orthosis and a walking-cane. Three persons scored a 4 on the FAC, indicating an ability to walk independently on level surfaces, but required help on uneven surfaces, stairs, or inclines. Participant characteristics are summarized in Table 1 for the participants that played Wii tennis (n = 7) and boxing (n = 8). Three participants were unable to play the tennis game, because of problems with timing of hitting the ball. A technical problem with the calorimeter invalidated VO_2 _data collection from 2 participants during boxing.

**Table 1:** Characteristics of study participants

	Tennis (n = 7)	Boxing (n = 8)
Men/women (n)	3/4	5/3
Age (yrs)	48 (33-68)	56 (33-74)
Mass (kg)	84.5 ± 19.7	81.9 ± 10.3
Handedness, right	7	8
Sum of skinfolds (mm)	91.9 ± 27.2	83.7 ± 15.9
% Body fat	35.6 ± 7.0	34.6 ± 5.8
Fat-free Mass (kg)	53.9 ± 10.6	53.5 ± 7.7
Height (cm)	171.9 ± 7.2	172.6 ± 7.5
Body mass index (kg/m^2^)	28.4 ± 5.4	27.5 ± 2.7
Time post-stroke (months)	34.3 (9-119)	18.6 (9-30)
Stroke severity: PACS/POCS	4/3	6/2
Affected side, right	4	6
MRC	4.1 (1-5)	4.3 (1-5)
MAS	0.4 (0-2)	0.5 (0-2)
Balance (BBS)	54.7 (52-56)	54.9 (52-56)
mRS	1.7 (1-3)	2.1 (1-3)

Values are mean ± standard deviations or mean (range). PACI = partial anterior circulation syndrome, POCI = posterior circulation syndrome; MRC = Medical Research Council; MAS = Modified Ashworth scale; BBS = Berg Balance Scale; mRS = modified Rankin Scale.

**Figure 2:**
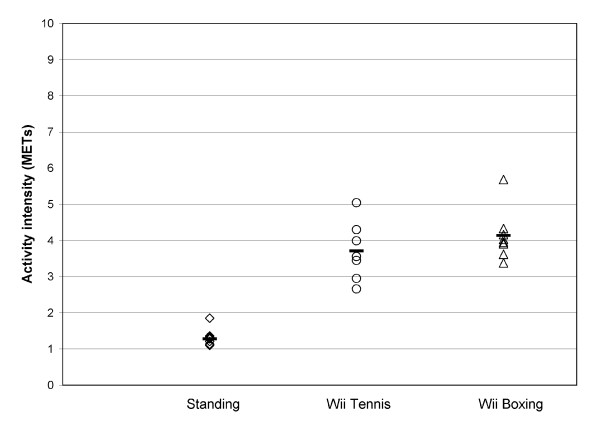
**Participants' mean energy expenditure while standing and during Wii Sports tennis (n = 7) and boxing (n = 8) game play**. Horizontal dashes indicate group mean energy expenditure. METs = metabolic equivalents.

The mean (SD) VO_2 _during sitting was 3.0 (0.8) ml/kg/min for the participants who played tennis and 2.9 (0.7) ml/kg/min for those who played boxing. For standing the mean VO_2 _was 3.6 (1.1) ml/kg/min for the participants who played tennis and 3.8 (0.9) ml/kg/min for those who played boxing. Compared with sitting, VO_2 _was 30% higher when standing for the tennis group and 31% higher for the boxing group (P = 0.01). Wii Sports tennis increased the VO_2 _267% compared with sitting (P = 0.02), and 205% compared with standing (P = 0.02). Wii Sports boxing increased VO_2 _310% compared with sitting (P = 0.01), and 213% compared with standing (P = 0.01). Energy expenditure was higher for Wii boxing (4.1 METs) compared to Wii tennis (3.7 METs); however, this difference was not significant (P = 0.50) (Table 2). For all participants, the energy expenditure was ≥ 3 METs during boxing (range 3.4 - 5.7 METs) (Figure 2). Only one participant had energy expenditure < 3 METs during tennis (range 2.7 - 5.0 METs). The mean perceived exertion was rated higher for Wii Sports boxing (5.3) than for tennis (4.1) (P = 0.034) (Table 2). The individual perceived exertion rates and MET values for tennis and boxing are presented in table 3.

**Table 2:** Cardiorespiratory variables, energy expenditure, and perceived exertion of the 15 minutes Wii game play

	Tennis (n = 7)	Boxing (n = 8)	*P*
VO_2 _(ml/min)	891.6 (249.1)	980.1 (319.2)	0.345
VO_2 _(ml/kg/min)	11.0 (3.9)	11.9 (3.3)	0.345
VO_2 _(ml/FFM/min)	17.0 (5.2)	18.1 (4.4)	0.345
HR (beats/min)	96.8 (14.7)	106.1 (20.0)	0.225
V_E _(L/min)	25.7 (4.7)	33.2 (10.3)	0.225
RER	0.91 (0.06)	0.95 (0.07)	0.500
Energy expenditure (METs)	3.7 (0.8)	4.1 (0.7)	0.500
Perceived exertion	4.1 (1.2)	5.3 (2.7)	0.034

Values are mean ± standard deviation

VO_2 _= oxygen uptake

FFM = fat free mass

HR = heart rate

V_E _= pulmonary ventilation

RER = respiratory exchange rate

METs = metabolic equivalents

**Table 3:** Individual values for energy expenditure and rating of perceived exertion of the 15 minutes Wii game play

Patient	Age	Gender	Tennis METs	Tennis RPE	Boxing METs	Boxing RPE
1	33	m	2.7	3	4.3	4
2	57	v	4.3	3	3.4	4
3	57	m	na	na	4.2	8
4	74	m	na	na	4.0	0.5
5	70	m	na	na	5.7	3
6	35	v	3.4	5	na	na
7	68	v	3.6	4	3.9	9
8	45	m	3.0	5	4.0	6
9	44	v	4.0	3	3.6	4
10	52	m	5.0	6	na	na

Mean	53.6	na	3.7	4.1	4.1	4.8
Median	54.5	na	3.6	4.0	4.0	4.0
Range	33-74	na	2.7-5.0	3-6	3.4-5.7	0.5-9

METs = metabolic equivalents

RPE = rating of perceived exertion

na = data are not available/applicable

## Discussion

The aim of this study was to determine energy expenditure during Wii Sports tennis and boxing game play in chronic stroke patients. Our results show that the energy expenditure during Wii Sports boxing and tennis was ≥ 3 METs for all except for one participant during tennis.

According to the ACSM/AHA guidelines for adults, the energy expenditure in these chronic stroke patients was sufficient to improve and maintain health [20]. Therefore, Wii Sports tennis and boxing may be useful to increase activity levels and to promote a healthy lifestyle in patients with stroke. The recommended activity dose for healthy adults is moderate-intensity physical activity (3-6 METs) for a minimum of 30 minutes on five days each week or vigorous-intensity physical activity (> 6 METs) for a minimum of 20 minutes on three days each week [20]. Thirty minutes of moderate-intensity Wii activities could be attained by playing several 10-minute games of tennis or boxing. Alternatively, combinations of Wii Sports game play with other moderate-intensity activities (e.g., walking, dancing) could also be used to meet the ACSM/AHA target levels.

Defining aerobic intensity in absolute terms might not be appropriate for older adults and adults with chronic conditions, because they often have low fitness levels [4-6,41]. For older adults with low fitness levels, ACSM/AHA recommends the modified Borg scale to measure intensity of physical activity [41]. On this 10-point scale, a 5 to 6 is considered moderate-intensity activity and a 7 to 8 is considered vigorous-intensity physical activity. Six of our participants were 'older adults' (as defined by the ACSM/AHA guidelines; i.e. age≥ 65 years or age 50 to 64 years with clinically significant chronic conditions) of whom 3 scored ≥7 on the modified Borg scale for boxing but had corresponding MET values < 6. Because more intense activities are presumed to provide greater health benefits, these 3 participants might have greater health benefits than expected from their MET values [20]. Seven participants rated their perceived exertion < 5 but had MET values > 3, possibly as a result of the heterogeneity of fitness levels in our sample. Because of the possible differences in fitness levels and because the Borg scale is a subjective measure, we prefer to use the objective measured MET values.

Although expected, given the results from previous studies [42,43], the energy expenditure during Wii boxing was not significantly higher than during Wii tennis. Graves et al. [43] found higher energy costs in healthy persons during Wii boxing compared with Wii tennis. They suggested that this resulted from the nature of the boxing game encouraging the use of both arms, as non-dominant limb activity was significantly greater than during tennis. Our participants were limited from using their affected arm during boxing, which might explain why differences in energy expenditure between boxing and tennis were not found.

Stroke survivors commonly have impaired balance while standing, which might induce relatively large energy costs during standing compared with sitting. The mean energy expenditure during standing (1.3 METs) was relatively low compared with energy expenditure during game play. Additionally, the MET intensities for standing in our sample were comparable with the MET intensities in able-bodied persons for standing quietly reported by Ainsworth et al. [8]. Therefore, the increased energy expenditure during Wii Sports resulted primarily from game play.

All participants were able to play Wii boxing without extensive instruction and training. Problems with timing of hitting the ball limited 3 participants from playing Wii tennis, most likely resulting from stroke-induced deficits in spatial and temporal coordination or reduced motor response from advanced age [44,45]. Holding the Wii remote and Nunchuk was not possible for one person because of severe spasticity in the fingers. This person could have played the games by simply fixating the Wii remote to the hand (e.g. using a latex band); however, additional assistance would be required to push the Wii remote buttons for starting and stopping the game. For safety reasons supervision is needed when a stroke patient with balance problems plays Wii games while standing. We found no adverse effects, (e.g. nausea or dizziness, repetition injuries, and epileptic seizure), which would limit the applicability of active video games as an exercise tool for stroke patients [21,46]. However, two patients felt temporarily very fatigued after boxing (perceived exertion of 8 and 9) and had mild soreness of the shoulder. Given current literature, repetition injuries seem to be the main concern when playing exergames [46-48]. Especially for stroke patients with musculoskeletal problems (e.g. muscle weakness and impaired joint stability), supervision is important to avoid exercise overdose.

This is a proof-of-principle study with a small convenience sample evaluating one 15-minute session of 2 Wii Sports games. The measurements were performed in a laboratory setting with two researchers observing the participant. However, we do not expect energy expenditure to differ substantially from home use because participants were instructed to play the games at their preferred intensity and manner, without encouragement by the researchers. Also, the participants wore a calorimeter face-mask, which differs from home use of the Wii; however, these caused no observable interference with game play. Nevertheless, we are aware that the participants were engaged in an experimental study; therefore, their behaviour will not necessarily be the same when playing Wii tennis and boxing at home. Larger prospective studies are needed to determine the effectiveness and potential side-effects of Wii game play for maintaining and improving health in chronic stroke patients. Also, future studies should focus on optimisation of exergames regarding hardware and software, so that a wide variety of stroke patients can enjoy and hopefully benefit from exergaming.

## Conclusions

In general, Wii Sports tennis and boxing were performed by nearly all chronic stroke patients in this study at sufficient intensity to maintain and improve health. Further research is needed to determine the effectiveness of exergames in improving daily activity levels and cardiorespiratory fitness among stroke survivors. For this it is important to assess which stroke patient most likely will benefit from playing exergames.

## List of abbreviations

none

## Competing interests

The authors declare that they have no competing interests.

## Authors' contributions

HLH and RJBE contributed to the design and methodology of the study. MFSK and HLH contributed to the acquisition of the data. HLH, MFSK and RJBE analyzed the data, and HLH, GMR, HJS and RJBE interpreted the data. All authors read and approved the manuscript.
